# Green hospitals: Mitigating water footprint and greenhouse gas emissions through sustainable menu planning in Turkish state university hospitals

**DOI:** 10.1002/fsn3.4244

**Published:** 2024-06-07

**Authors:** Betül Oruçoğlu, Mehmetcan Kemaloğlu, Emine Kemaloğlu

**Affiliations:** ^1^ Department of Nutrition and Dietetics Afyonkarahisar Health Sciences University Afyonkarahisar Turkey; ^2^ Department of Nutrition and Dietetics Ağrı İbrahim Çeçen University Ağrı Turkey

**Keywords:** Green hospitals, greenhouse gas emissions, hospital meal menu, sustainable diets, water footprint

## Abstract

Considering the importance of sustainable nutrition, it is important that hospitals' meal menus are planned to ensure the lowest possible environmental footprint. In this study, we aimed to evaluate the environmental effects of hospital menus and the changes that may occur when these menus are planned according to the Turkey Dietary Guidelines and Mediterranean diet recommendations. In this context, first, the yearly environmental footprints of the standard meal menus of the state university hospitals in Turkey (*n* = 42), including water footprint (WF) and greenhouse gas emission (GHGE) values, were determined. Second, changes in the environmental footprint as a result of arranging the standard meal menus of state university hospitals according to the Turkey Dietary Guidelines and Mediterranean nutritional models were evaluated. It was determined that the average WF and GHGE values of hospital menus were 137,280 ± 18537.2 L/month and 140.0 ± 18.4 kg CO_2_‐eq/month, respectively. Adjusting state university hospitals' standard meal menus according to Turkey Dietary Guidelines and Mediterranean nutritional models reduced WF by 24.8% to 103206.7 L/month and 37.8% to 85420.5 L/month, and GHGEs by 31.7% to 95.5 kg CO_2_‐eq/month and 49% to 71.3 kg CO_2_‐eq/month, respectively. In addition, it was determined that hospital meal menus planned according to the Turkey Dietary Guidelines and the Mediterranean nutritional model contained lower saturated fat and cholesterol and higher dietary fiber. In conclusion, planning hospital menus according to the Turkey Dietary Guidelines and Mediterranean nutritional recommendations can reduce the environmental footprint of hospital food services.

## Graphical abstract

The environmental footprints of Turkish state university hospital menus were analyzed. Adapting to dietary guidelines significantly reduces environmental impacts. Sustainable hospital menus can guide the public toward eco‐friendly diets.

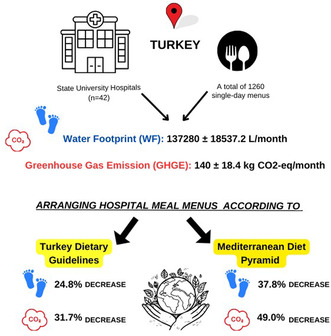

## 1 INTRODUCTION

Among numerous global environmental challenges, climate change is recognized as the greatest global health threat of the 21st century, carrying serious implications for human health through extreme weather events, changes in infectious disease patterns, increased food insecurity, and degradation of air and drinking water quality (Costello et al., 2009; Watts et al., 2015, 2018). Changes in people's food consumption patterns featuring consumption of highly processed and animal‐based foods and moving away from healthier and more traditional plant‐based eating patterns cause the current food system to increase both the burden of non‐communicable chronic diseases and the severity of the climate crisis (Barbour et al., 2022; IPCC, 2021; Willett et al., 2019). Considering that food systems disrupt natural ecosystems by generating large amounts of greenhouse gas emissions (GHGE), depleting water resources, and causing land degradation and loss of biodiversity, the importance of transitioning to a healthy and more climate‐friendly food system is indisputable (IPCC, 2021; The Lancet Planetary Health, 2019; Willett et al., 2019).

Sustainable dietary models are defined as nutritionally adequate, economically appropriate, culturally acceptable, and environmentally‐friendly models (Vieux et al., 2018). Achieving ecologically sustainable food requires coordinated, multi‐sectoral changes at local, national, and global levels aimed at improving food consumption patterns (United Nations, 2015; Watts et al., 2018). Many studies have shown that transitioning to a healthier dietary model can provide numerous environmental benefits, including water savings (Blas et al., 2016, 2018; Vanham et al., 2013, 2018). It has been reported in several studies that a reduction in total dietary water footprint (Chaudhary & Krishna, 2019; Harris et al., 2020; Springmann et al., 2018; Tompa et al., 2022) and GHGE (Clune et al., 2017; Drew et al., 2020; The World Bank, 2017) can be achieved by transforming individuals' dietary patterns into healthy ones that comply with national nutritional guidelines. It is also advocated by many researchers that the Mediterranean diet, which contains fewer animal‐derived products compared to the Western‐style diet, has a lower environmental impact, i.e., less carbon and water footprint and ecological impact (Aboussaleh et al., 2017; Berry et al., 2015; Burlingame & Dernini, 2011; Capone et al., 2013; Dernini et al., 2013, 2017; Dernini & Berry, 2015; Kemaloglu et al., 2023; Shahar et al., 2023; Vieux et al., 2018).

Hospital services also include catering services to provide food to patients, staff, and visitors. More than 10 million patients received inpatient treatment in hospitals in Turkey in 2020. Approximately 1.5 million of these patients received care in state university hospitals. Additionally, it was reported that nearly 180 thousand healthcare staff have been working under different titles in state university hospitals in 2020 (The Ministry of Health of Turkey, 2021). Thousands of meals are served to patients, staff, and visitors in hospitals that operate 24 h a day and 365 days a year, requiring massive amounts of resources, including water, energy, and land, to produce the foods served in these meals. An environmental footprint is created at every step of this food procurement process (Carino et al., 2020; Melikoglu, 2020).

Therefore, professionals aiming to improve the environmental impact of healthcare services need to focus more on the sustainability of the food procurement process (Vidal et al., 2015). However, there are a limited number of studies on the food groups that will render hospital meal menus environmentally sustainable (Carino et al., 2020; Vidal et al., 2015). In light of this information, this study was carried out to: (i) determine the yearly environmental footprint of the standard meal menus of the state university hospitals in Turkey and (ii) evaluate the changes in the environmental footprint (WF and GHGE) as a result of adjusting the state university hospitals' meal menus according to the Turkey Dietary Guidelines and Mediterranean nutritional models.

## 2 MATERIALS AND METHODS

### 2.1 Research design

Data collection occurred from November 2021 to August 2022. During this time period, a one‐year retrospective (2021) menu was requested from hospitals. As of 2020, 50 state university hospitals actively provide services in Turkey (The Ministry of Health of Turkey, 2021). Of these hospitals, eight hospitals that refused to share information were excluded from the study. In the end, the yearly meal menus of 42 state university hospitals were evaluated within the scope of the study. The stratified sampling method was used to ensure homogeneity in terms of geographical regions. Accordingly, each of the 12 service regions defined in the Health Statistics Yearbook of the Ministry of Health of the Republic of Turkey (Mediterranean: R1; Western Anatolia: R2; Western Black Sea: R3; Western Marmara: R4; Eastern Black Sea: R5; Eastern Marmara: R6; Aegean Region: R7; Southeastern Anatolia: R8; Istanbul Region: R9; Northeastern Anatolia: R10; Middle Eastern Anatolia: R11; Central Anatolia: R12) (The Ministry of Health of Turkey, 2021) was accepted as a stratum, and the calculated number of samples was distributed to these strata by the “proportional distribution” method.

The following equation was used to calculate the number of samples:

$$n=\frac{Nt^{2}\sum \left(P_{i}{\mathrm{CV}}_{i}\right)}{NE^{2}+t^{2}\sum \left(P_{i}{\mathrm{CV}}_{i}\right)}$$

where *n*: number of samples to be included in the study, *N*: total number of samples, *P*_*i*_: ratio of the number of samples in the *i*th stratum to the total number of samples, CV_*i*_: variability of the *i*th stratum, and *E*: sampling error percentage.

Since there was no information about the variation of the population, the coefficient of variation (*CV*) was accepted as 0.50, and the sampling error percentage (*E*) was accepted as 0.10 in the calculations. The calculated number of samples (*n*) was distributed proportionally to the strata (regions), and the number of samples for each region (*n*_*i*_) was calculated. The samples distributed to the regions were further distributed to the sub‐regions proportionally, taking into account the number of hospitals in the (*n*_*i,j*_) sub‐regions.

The potential number of days calculated by systematic sampling of the yearly meal menus of state university hospitals was 30 days. Accordingly, the sampling interval was found to be 12.17 by dividing 365 by 30 and was accepted as approximately 12 days. Afterwards, a number between 1 and 12 was randomly selected, and a sampling calendar was created at 12‐day intervals from the selected day of the year until the end of the year. Accordingly, at first, the number ‘4’ was randomly selected. Hence, the sample pertaining to the 4th day of the year (January 4th) was accepted as the first sample, and then a total of 30 samples were determined for 30 days, including the samples pertaining to the 16th, 28th, 40th, 52nd, …, 352nd days.

The number of healthcare personnel (with different titles) working in the university hospitals included in the research is approximately 150 thousand, and the number of hospital beds is approximately 37 thousand.

### 2.2 Standard meal menus of the state university hospitals

A total of 1260 single‐day menus, including 30 menus from 42 hospitals, were analyzed. Nutrient contents and environmental footprint analyses of state university hospitals were conducted for the food groups included in the breakfast, lunch, and dinner of each state university hospital included in the study. Standard hospital diets and meals served to hospital staff were included in the analyses. Therapeutic diets and meals served to pediatric patients were excluded from the study. Standard food recipes were used to evaluate the contents of the menus (both nutritional content and environmental footprint). In addition to 1‐year meal menus, hospitals were also requested to provide the standard meal recipes they used during meal preparation. Standard recipes specify the serving size of each dish and the name and quantity of ingredients in the serving, and the study's calculations were made using the amounts included in these recipes.

The features of standard hospital menus offered in hospitals in Turkey by the Inpatient Treatment Institutions Operation Regulation (The Ministry of Health of Turkey, 1983) are defined as follows:

- Menus consist of three meals: breakfast, lunch, and dinner.
- A maximum of three types of breakfast items other than bread, tea, or milk are served for breakfast (cheese and eggs are not served together).
- Lunch and dinner are three bowls. Care should be taken to include meat, vegetables, starchy food items, fruits, or desserts in the preparation. Two meat, two starchy, or two vegetable dishes should not be served in the same meal. When two sweet and/or starchy meals are given on the same day, dumplings are not given.

The groups of foods, recipes, and daily maximum amounts of these dishes (according to the Inpatient Treatment Institutions Operating Regulations) are given in Table S1. Since bread consumption was not reported, the amount of bread was not included in the calculations. Nutrition Information Systems Package Software (BeBiS) version 8.2 was used to calculate the energy and nutrient contents of the state university hospitals' meal menus. WF and GHGE analyses of state university hospitals' meal menus were conducted on the basis of 12 geographical regions. The analysis results were reported as the average of regions (AOR).

### 2.3 Nutritional models used in planning alternative meal menus to the standard meal menus of state university hospitals

Based on the findings of relevant studies in the literature (Aboussaleh et al., 2017; Berry et al., 2015; Burlingame & Dernini, 2011; Capone et al., 2013; Chaudhary & Krishna, 2019; Dernini et al., 2013, 2017; Dernini & Berry, 2015; Harris et al., 2020; Shahar et al., 2023; Springmann et al., 2018; Tompa et al., 2022; Vieux et al., 2018), two nutritional models, i.e., Turkey Dietary Guidelines and Mediterranean nutritional models, were analyzed within the scope of the study. The alternative menus determined based on Turkey Dietary Guidelines (TDG) and Mediterranean (MED) nutritional models were comparable to AOR in terms of average daily energy content (AOR: 1549.5 ± 193.8 kcal/day; TDG: 1546.2 kcal/day; MED: 1553.0 kcal/day). The Turkey Dietary Guidelines is a food‐based national nutrition guide that provides science‐based recommendations about the foods and beverages recommended for Turkish people to consume in order to improve their health, reduce the risk of non‐communicable chronic diseases, and meet their nutritional needs (The Ministry of Health of Turkey, 2022). TDG was created according to the recommendations of the Turkey Dietary Guidelines given below:

- Energy components: 45%–60% carbohydrates, 20%–35% fats, and 10%–20% proteins
- Animal origin foods: Red meat: <50 g/day; fish/seafood: ≥2 servings/week; dairy products: 3 servings/day; and chicken egg: 2 servings/week
- Plant origin foods: Fruits: 2–3 servings/day; vegetables: 2–3 servings/day; legumes: 2–3 servings/week; and bread/pasta/rice/couscous/other cereals: 3–7 servings/day
- Oils and fats: one unit of fat, one unit of any vegetable oil, and one and a half or two units (1.5 or 2 units) of olive oil: 1; 1; 1.5/2. The intake of fat should be kept at a minimum level (energy <10%)
- Sugar and sweets: <10% of the energy amount required daily

The Mediterranean diet model encourages the consumption of fresh plant‐based foods and fish, moderate consumption of meat and dairy products, and the use of olive oil as the main oil in the preparation of meals (Bach‐Faig et al., 2011). MED was created according to the recommendations of the new Mediterranean diet pyramid given below:

- Energy components: 45%–60% carbohydrates, 20%–35% fats and 10%–20% proteins
- Animal origin foods: red meat: <2 servings/week; white meat: 2 servings/week; fish/seafood: ≥2 servings/week; dairy products: 2 servings/day (preferably low fat); chicken egg: 2–4 servings/week
- Plant origin foods: fruits: 1–2 servings/every main meal; vegetables: ≥2 servings/every main meal; legumes: ≥2 servings/week; bread/pasta/rice/couscous/other cereals: 1–2 servings/every main meal (preferably whole grain)
- Oils and fats: olive oil (every main meal)
- Sugar and sweets: ≤2 servings/week

### 2.4 Evaluation of the water footprints of the standard and alternative state university hospital meal menus

The reference values reported in Mekonnen and Hoekstra's study (Mekonnen & Hoekstra, 2011), which contains weighted average WF data of many food products in different countries, including Turkey, were used to calculate the WF values of the menus investigated within the scope of this study. In WF calculations, only the WFs of the raw products were taken into account, while the WFs pertaining to the water used for cooking were not. The WF values for seafood and saltwater fish products were considered to be zero. The weighted average WF values of the food products included in the menus were multiplied by their respective amounts. All three types of WF, i.e., blue, green, and gray, were calculated for AOR, TDG, and MED.

WF values were calculated for each food in standard meal recipes as follows:

Egg WF = water footprint of egg (m^3^/g^−1^) × average egg consumption (g).

### 2.5 Evaluation of greenhouse gas emissions from the standard and alternative state university hospital meal menus

Since there is no national data on GHGE and carbon footprint values of foods produced in Turkey, the GHGE values given in kg/product in Clune et al.'s study (Clune et al., 2017) were taken as references in calculating the GHGE values of regions AOR, TDG, and MED.

GHGE values were calculated for each food in standard meal recipes as follows:

Egg GHGE = greenhouse gas emissions of egg (kg CO_2_‐eq/g) * average egg consumption (g).

Foods were classified into 12 groups: (Watts et al., 2018) milk and dairy products; (Costello et al., 2009) red meat; (Watts et al., 2015) poultry; (Willett et al., 2019) eggs; (Barbour et al., 2022) cereals; (IPCC, 2021) legumes and pulses; (The Lancet Planetary Health, 2019) vegetables; (Vieux et al., 2018) fruits; (United Nations, 2015) vegetable oils and fats; (Blas et al., 2016) nuts; (Blas et al., 2018) sugar; and (Vanham et al., 2013) others (ingredients that added color, flavor, and texture to meals like vinegar, tomato extract, a meat tenderizer, and all kinds of salts). Detailed information about the food groups and quantities included in this study is shown in Table S2.

### 2.6 Statistical analysis

The descriptive statistics obtained from the collected data were tabulated as mean ± standard deviation values and median with minimum and maximum values in the case of continuous (numerical) variables depending on their normal distribution characteristics and as numbers and percentage values in the case of categorical variables. Normal distribution characteristics of the numerical variables were analyzed using Shapiro–Wilk, Kolmogorov–Smirnov, and Anderson–Darling tests. Statistical analyses were conducted using Jamovi project 2.3.24.0 (Jamovi, version 2.3.24.0, 2023, retrieved from https://www.jamovi.org) and JASP 0.17.1 (Jeffreys' Amazing Statistics Program, version 0.17.1, 2023, retrieved from https://jasp‐stats.org) software packages. Probability (p) statistics of ≤0.05 were deemed to indicate statistical significance.

### 2.7 Ethical considerations

This study was conducted in accordance with the principles outlined in the Declaration of Helsinki. The study protocol was approved by the Ethics Committee of Ağrı İbrahim Çeçen University (Approval Number: 346; Approval Date: November 25th, 2021) prior to the conduct of the study.

## 3 RESULTS

Energy, macronutrient, and micronutrient contents of regions AOR, TDG, and MED are given in Table 1. Accordingly, the mean daily energy content of AOR, TDG, and MED were 1549.5 ± 193.8 kcal/day, 1546.2 kcal/day, and 1553.0 kcal/day, respectively. Additionally, the mean percentages of energy sourced from saturated fatty acids in the average daily energy content of AOR, TDG, and MED were 18.0% ± 1.8%, 10.8%, and 7.9%, respectively. The mean percentages of plant‐derived protein in the protein content of AOR, TDG, and MED were 31.6% ± 4.7%, 43.4, and 47.2, respectively. The mean dietary fiber contents of AOR, TDG, and MED were 16.4 ± 2.7 g/day, 31.2 g/day, and 40.1 g/day.

**TABLE 1:** Energy, macronutrient, and micronutrient contents of hospital menus and diet models.

	Energy (kcal/day)	Carbohydrates (%E)	Protein (%E)	Plant‐based protein (%protein)	Animal‐based protein (%protein)	Fat (%E)	Saturated fatty acids (%E)	Dietary fiber (g/day)	Cholesterol (mg/day)
R1	1499.6 ± 118.6	35.2 ± 2.4	18.6 ± 0.6	31.0 ± 5.1	69.0 ± 5.1	45.8 ± 2.8	18.5 ± 1.1	16.0 ± 2.9	298.6 ± 27.0
R2	1594.3 ± 267.8	36.4 ± 2.9	19.7 ± 1.4	29.3 ± 4.0	70.7 ± 4.0	44.0 ± 2.1	16.8 ± 1.1	16.6 ± 3.5	340.7 ± 59.0
R3	1610.1 ± 149.7	37.7 ± 0.6	19.0 ± 1.7	32.5 ± 4.8	67.5 ± 4.8	43 ± 2.0	16.4 ± 1.5	19.0 ± 1.6	347.3 ± 24.2
R4	1466.9 ± 215.1	46.3 ± 21.8	22.3 ± 8.5	38.1 ± 4.1	62.0 ± 4.1	56.0 ± 20.0	18.6 ± 1.8	17.1 ± 3.3	250.4 ± 54.2
R5	1745.3 ±	37.0 ±	19.0 ±	33.5 ±	66.6 ±	44.0 ±	17.4 ± .	20.1 ±	319.2 ±
R6	1824.3 ± 267.1	34.3 ± 2.1	18.3 ± 2.5	32.1 ± 5.2	67.9 ± 5.2	47.7 ± 4.1	19.2 ± 3.7	16.9 ± 3.1	389.6 ± 91.2
R7	1586.67 ± 116.7	37.2 ± 1.1	17.8 ± 1.5	32.8 ± 4.5	67.2 ± 4.5	45.0 ± 1.2	17.6 ± 1.3	17.4 ± 2.4	311.9 ± 17.7
R8	1625.1 ± 153.8	36.5 ± 2.1	19.0 ± 1.4	30.0 ± 1.1	70.0 ± 1.1	45.0 ± 1.4	16.6 ± 1.0	17.6 ± 0.7	418.2 ± 139.4
R9	1492.8 ± 146.1	33.6 ± 2.6	18.8 ± 0.5	37.3 ± 5.0	71.6 ± 5.0	47.6 ± 2.2	19.5 ± 2.0	14.5 ± 2.2	293.4 ± 34.6
R10	1489.2 ± 7.2	34.5 ± 3.5	19.5 ± 3.5	27.8 ± 3.2	72.2 ± 3.2	46.0 ± 0.0	18.2 ± 0.2	14.3 ± 1.2	335.6 ± 52.6
R11	1373.6 ± 185.8	35.5 ± 0.7	19.0 ± 2.8	32.1 ± 4.8	68.0 ± 4.8	45.5 ± 3.5	17.8 ± 2.3	14.9 ± 0.5	315.0 ± 1.5
R12	1377.8 ± 63.1	38.0 ± 2.7	17.3 ± 0.6	33.6 ± 2.8	66.4 ± 2.8	44.3 ± 2.9	19.0 ± 1.2	14.5 ± 0.6	251.8 ± 25.7
AOR	1549.5 ± 193.8	36.9 ± 7.1	19.1 ± 2.9	31.6 ± 4.7	68.4 ± 4.7	46.3 ± 6.7	18.0 ± 1.8	16.4 ± 2.7	317.3 ± 62.2
TDG	1546.2 ±	46.4 ±	19.7 ±	43.4 ±	56.6 ±	34.0 ±	10.8 ±	31.2 ±	224.4 ±
MED	1553.0 ±	44.6 ±	19.6 ±	47.2 ±	52.8 ±	35.9 ±	7.9 ±	40.1 ±	213.7 ±

	Calcium	Iron	Vitamin A	Zinc	Vitamin B12	Vitamin C	Vitamin K	Vitamin E	Thiamine	Riboflavin	Niacin
R1	635.8 ± 26.4	9.9 ± 1.0	968.0 ± 261.1	10.3 ± 0.9	6.2 ± 1.7	92.1 ± 17.2	94.7 ± 28.4	15.3 ± 1.6	0.9 ± 0.1	1.4 ± 0.1	29.5 ± 3.1
R2	609.8 ± 99.1	10.9 ± 1.6	1434.6 ± 371.9	11.1 ± 1.3	7.9 ± 2.9	91.4 ± 16.7	122.9 ± 18.2	15.8 ± 3.0	0.9 ± 0.2	1.5 ± 0.1	33.5 ± 4.7
R3	626.4 ± 61.7	11.3 ± 0.5	1011.0 ± 199.4	11.4 ± 1.0	6.6 ± 0.8	118.8 ± 24.2	109.7 ± 38.5	17.0 ± 1.4	1.0 ± 0.1	1.4 ± 0.1	32.3 ± 1.1
R4	660.2 ± 169.9	9.8 ± 1.9	1217.9 ± 322.6	9.9 ± 1.9	6.2 ± 1.7	83.3 ± 9.2	104.8 ± 15.3	11.5 ± 3.5	0.8 ± 0.2	1.4 ± 0.4	26.5 ± 4.8
R5	680.1 ± .	11.5 ± .	1426.6 ± .	12.3 ± .	7.1 ± .	144.7 ± .	166.7 ± .	17.4 ± .	1.1 ± .	1.5 ±	34.6 ± .
R6	704.9 ± 33.3	11.3 ± 0.8	1377.9 ± 322.1	12.7 ± 0.5	8.6 ± 2.2	86. ± 27.7	94.8 ± 34.2	16.8 ± 1.4	0.9 ± 0.1	1.7 ±	33.6 ± 1.6
R7	642.4 ± 59.0	10.4 ± 0.7	1146.1 ± 253.5	10.5 ± 1.0	6.0 ± 1.2	105.6 ± 19.4	148.3 ± 50.8	15.9 ± 2.1	0.9 ± 0.1	1.4 ± 0.1	28.5 ± 3.0
R8	679.3 ± 149.7	11.1 ± 1.0	1017.6 ± 68.6	12.0 ± 0.3	7.2 ± 0.4	125.9 ± 12.6	114.9 ± 14.4	17.5 ± 0.6	0.9 ± 0.1	1.6 ± 0.4	31.5 ± 1.9
R9	664.5 ± 75.9	9.5 ± 1.1	1143.0 ± 319.1	11.0 ± 0.6	7.0 ± 1.7	82.0 ± 7.6	112.4 ± 16.5	12.4 ± 2.6	0.8 ± 0.1	1.4 ± 0.2	28.7 ± 2.1
R10	509.8 ± 80.0	10.5 ± 1.6	985.9 ± 6.6	10.7 ± 1.4	6.0 ± 0.6	85.0 ± 22.4	145.8 ± 0.5	15.0 ± 1.7	0.8 ± 0.1	1.3 ± 0.1	32.5 ± 6.9
R11	501.6 ± 107.2	9.4 ± 0.3	904.2 ± 138.7	9.6 ± 0.5	5.6 ± 0.1	97.6 ± 10.0	109.5 ± 24.0	13.2 ± 1.0	0.8 ± 0.0	1.2 ± 0.1	26.9 ± 1.1
R12	565.6 ± 67.5	8.7 ± 0.4	839.4 ± 208.4	9.8 ± 0.9	6.2 ± 1.1	100.9 ± 10.4	110.3 ± 31.2	12.5 ± 1.3	0.7 ± 0.6	1.2 ± 0.1	24.9 ± 1.2
AOR	628.0 ± 93.0	10.3 ± 1.3	1145.9 ± 275.1	10.8 ± 1.3	6.8 ± 1.8	96.5 ± 20.4	116.9 ± 31.1	14.8 ± 2.8	0.9 ± 0.1	1.4 ± 0.2	30.0 ± 1.1
TDG	946.1 ± .	12.1 ± .	1203.8 ± .	11.0 ± .	4.6 ± .	130.6 ± .	242.2 ± .	15.4 ± .	1.3 ± .	1.7 ±	31.4 ± .
MED	806.7 ± .	14.6 ± .	1954.8 ± .	9.9 ± .	4.9 ± .	209.7 ± .	562.6 ± .	15.5 ± .	1.4 ± .	1.5 ±	32.0 ± .

Abbreviations: %E, percentage of energy; AOR, average of regions; MED, Mediterranean diet; R, region; TDG, Turkey Dietary Guidelines.

### 3.1 Water footprints of the standard and alternative state university hospital meal menus

**FIGURE 1:**
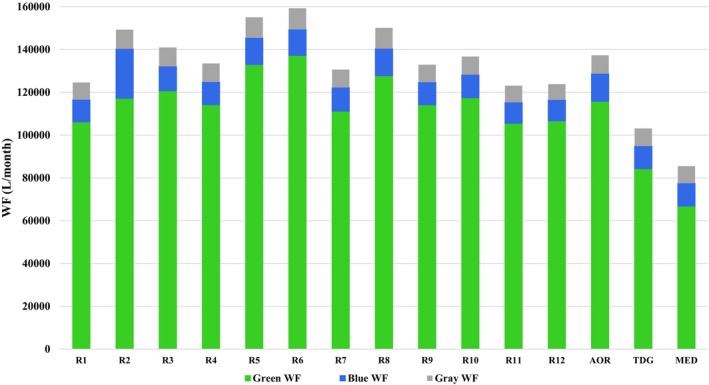
The total (green, blue, and gray) water footprint (WF) of hospital menus and diet models (L/month). AOR, average of regions; MED, Mediterranean diet; R, region; TDG, Turkey Dietary Guidelines.

The green, blue, gray, and total WF values of regions, AOR, TDG, and MED are given in Figure 1. Accordingly, mean green, blue, gray, and total WF values of AOR were 115756.6 ± 15012.3 L/month, 12941.7 ± 12527.9 L/month, 8581.9 ± 983.4 L/month, and 137,280 ± 18537.2 L/month, respectively. The three regions with the highest total WF values were R6 (159255.0 ± 16253.7 L/month), R8 (150086.5 ± 4312.8 L/month), and R2 (149260.9 ± 24872.3 L/month). In comparison, the mean total WF value of TDG was 103206.7 L/month and 24.8% less than the AOR, and the total WF value of MED was 85420.5 L/month and 37.8% less than the AOR (Figure 1).

### 3.2 Greenhouse gas emissions from the standard and alternative state university hospital meal menus

**FIGURE 2:**
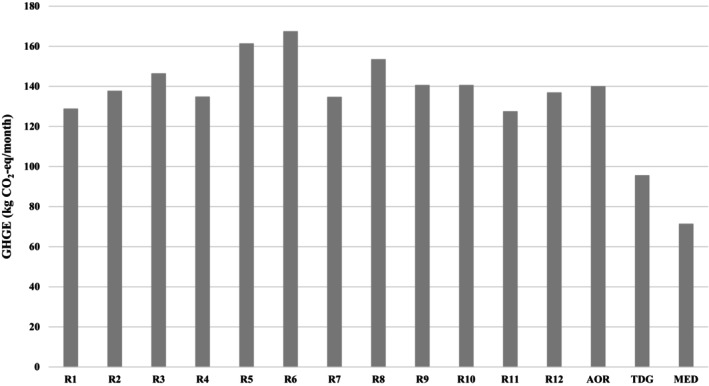
Greenhouse gas emission (GHGE) of hospital menus and diet models (g CO^2^‐eq per month). AOR, average of regions; MED, Mediterranean diet; R, region; TDG, Turkey Dietary Guidelines.

The GHGE values of regions, AOR, TDG, and MED are shown in Figure 2. The GHGE values of AOR, TDG, and MED were 140.0 ± 18.4 kg CO2‐equivalent/month, 95.5 kg CO2‐eq/month, and 71.3 kg CO2‐eq/month, respectively. Accordingly, TDG and MED had 31.7% and 49.0% less GHGE compared to AOR. Numerical data on the WF and GHGE values of hospital menus and alternative menus are given in Table S3.

### 3.3 Distribution of water footprints and greenhouse gas emissions of the standard and alternative state university hospital meal menus by the food groups

**FIGURE 3:**
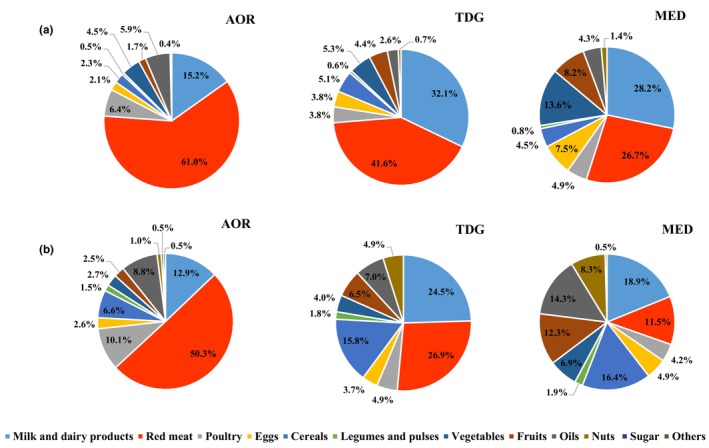
Distribution percentages of water footprint (WF) (a) and greenhouse gas emission (GHGE) (b) of hospital menus and diet models according to food groups. AOR, average of regions; MED, Mediterranean diet; R, region; TDG, Turkey Dietary Guidelines.

The WF values of AOR, TDG, and MED by the food groups are shown in Figure 3a. Accordingly, it was determined that the three food groups with the highest total WF values in AOR were red meat (61.0%), milk and dairy products (15.2%), and poultry (6.4%), respectively. Similarly, the food groups with the highest total WF values in TDG and MED were red meat and milk and dairy products. However, the third food group with the highest WF in both TDG and MED was vegetables.

The GHGE values of AOR, TDG, and MED by the food groups are shown in Figure 3b. Accordingly, it was determined that the three food groups with the highest total GHGE values in AOR were red meat (50.3%), milk and dairy products (12.9%), and poultry (10.1%), respectively. The food groups with the highest total GHGE values in TDG were red meat (26.9%), milk and dairy products (24.5%), and cereals (15.8%), respectively. The food groups with the highest total GHGE values in MED were milk and dairy products (18.9%), cereals (16.4%), and oils (14.3%), respectively.

**FIGURE 4:**
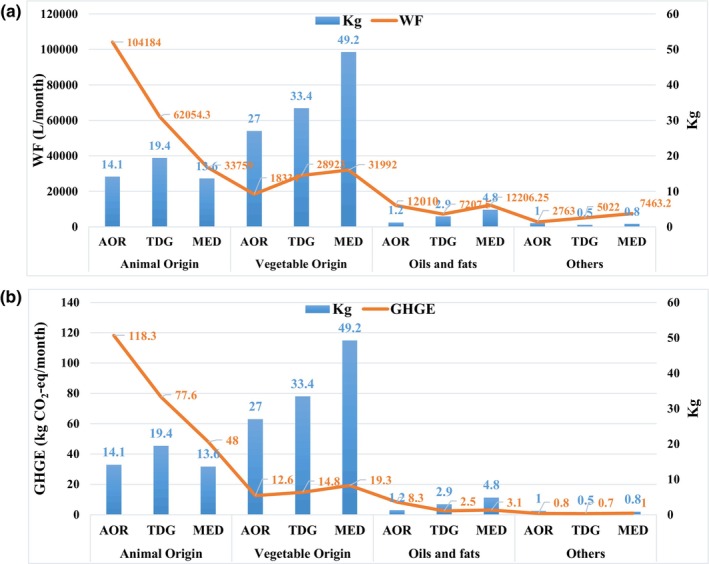
Relationship between food weight and water footprint (WF) (a) and greenhouse gas emission (GHGE) (b) by food groups of hospital menus and diet models. AOR, average of regions; MED, Mediterranean diet; R, region; TDG, Turkey Dietary Guidelines.

When food groups were compared in terms of quantity and WF per kilogram, it was seen that foods of animal origin had a higher WF value, considering the supply ratio of these foods in kilograms. The inverse situation between the amount of food groups in kg and WF is shown in Figure 4a. The same reverse situation was observed between the amount of food groups in kg and GHGE (Figure 4b).

## 4 DISCUSSION

This study was carried out to determine the environmental impacts of state university hospital's meal menus in Turkey. In addition, in line with the findings and recommendations of the relevant studies available in the literature (Aboussaleh et al., 2017; Berry et al., 2015; Burlingame & Dernini, 2011; Capone et al., 2013; Chaudhary & Krishna, 2019; Dernini et al., 2017; Dernini & Berry, 2015; Harris et al., 2020; Shahar et al., 2023; Springmann et al., 2018; Tompa et al., 2022; Vieux et al., 2018), the changes in the WF and GHGE values as a result of adjusting the standard meal menu of the state university hospitals according to TDG and Mediterranean nutritional models were determined. Consequently, the study's findings demonstrated that planning state university hospitals' meal menus based on TDG and Mediterranean nutritional models may reduce both WF and GHGE values.

### 4.1 The impact of hospital menus on environmental sustainability

The healthcare sector, which aims to protect and improve the general population's health, poses a series of threats to the sustainability of the environment due to the intense amount of energy it uses (Dhillon & Kaur, 2015; Eckelman & Sherman, 2018; Faezipour & Ferreira, 2013, 2018; Karliner et al., 2019; World Health Organization, 2010). Food production is one of the leading causes of environmental degradation due to high GHGE, water scarcity, water and air pollution, and biodiversity loss (Mekonnen & Gerbens‐Leenes, 2020; Milford & Kildal, 2019). Hospitals, one of the most important components of health systems, provide food service to employees, patients/families 24 h a day, 7 days a week, throughout the year (Eckelman & Sherman, 2018; GGHHA, 2015; Singh, 2019). The World Health Organization has reported that healthcare facilities can reduce their environmental footprint by making changes to their food and beverage service practices, including limiting the amount of meat in hospital meals, producing the meals served to patients and staff in‐house, purchasing local and organic products, etc., and helping prevent diseases by supporting access to food and nutrition (World Health Organization, 2010). Therefore, hospitals can model and promote health and sustainability through their food choices (NHS England, 2020). Details of the meal menus offered in hospitals in Turkey, such as the weekly frequency and amounts of meal groups, are specified in the Inpatient Treatment Institutions Operation Regulation (The Ministry of Health of Turkey, 1983). However, the relevant regulation only covers the maximum frequency and quantity of food groups that can be served on meal menus. For example, the maximum daily dose for milk and yogurt is indicated as 250 g, and the maximum weekly frequency of milk and yogurt is indicated as 7 (Table S1). In this study, the differences in the distribution of environmental footprints (both WF and GHGE) of hospital hospitals by region indicate that the Inpatient Treatment Institutions Operation Regulation should also focus on the environmental impacts of hospital food services. Determining food menus within a framework that is both healthy and respectful to the environment can prevent such differences in the environmental footprints of different hospitals affiliated with the same institution. Considering that the WF and GHGE values of menus are parallel to each other, it seems possible that correct menu planning principles can reduce both environmental footprints. The findings of our study also demonstrated that existing hospital meal menus can be changed to healthier (lower saturated fat and cholesterol, higher dietary fiber) (Table 1) meal menus with lower environmental footprints (Figures 1 and 2) if planned according to TDG and Mediterranean nutritional models, reducing the environmental burden caused by the healthcare system.

### 4.2 Dietary composition and environmental footprint

Dietary composition has a strong relationship with the environmental footprint. Diets high in energy, saturated fat, processed foods, added sugars, and red meat are thought to be associated with higher GHGE, land use, and water use compared to vegetable‐based diets (Fanzo & Davis, 2019). It has been reported that reducing meat consumption and increasing the supply of fish and plant‐based proteins such as beans, lentils, peas, and chickpeas may be an alternative to reducing the WF of menus offered without compromising nutritional quality (Nogueira et al., 2020). This study's findings indicated that AOR, TDG, and MED have comparable energy contents, yet AOR contains higher animal protein, lower vegetable protein, and higher saturated fat than TDG and MED; consequently, AOR has higher WF and GHGE compared to TDG and MED.

Animal origin foods are cited as the main cause of the environmental food‐related footprint (Hoekstra, 2011; Röös et al., 2014). Strasburg and Jahno (Strasburg & Jahno, 2017) demonstrated a positive association between the amount of meat and WF of food offered in university restaurants and recorded an average of 2 L of WF for each meal prepared, with the highest contribution coming from beef. The animal food group accounted for 77.9% of the WF, with beef and chicken being responsible for 62.2% of this value. On the other hand, those of vegetable origin provided the highest amount in kilograms of food (65.5% of the total) with a WF of 21.2%. Hatjiathanassiadou et al. (Hatjiathanassiadou et al., 2019) found WF values per capita of 2752.4 L for traditional lunch menus and 1113.9 L for vegetarian menus. The difference in these values was due to the use of foods of animal origin, mainly beef. These results are similar to those found in the present study. In this study, it was determined that the share of red meat and poultry, the food group with the highest mean environmental footprint value, in the total WF value decreased by 32.6% and 53.1%, respectively, as a result of adjusting the standard meal menu of the state university hospitals according to TDG and MED nutritional models (not shown in the table). When food groups were compared in terms of quantity and WF per kilogram, it was seen that foods of animal origin had a higher WF and GHGE value, considering the supply ratio of these foods in kilograms (Figure 4).

There are many studies in the literature that show that reducing meat consumption is effective in reducing the environmental impact of the diet (Aleksandrowicz et al., 2016; Biesbroek et al., 2014; Geibel & Freund, 2023; Perignon et al., 2017). In this study, it was determined that the share of red meat in total GHGE decreased by 46.5% and 77.1% as a result of adjusting the standard meal menu of the state university hospitals according to TDG and MED (not shown in the Table), and our findings are compatible with the literature.

The concept of diet sustainability refers to the nutritional adequacy, cultural acceptability, economic availability, and sustainable environmental impact of diets (Vieux et al., 2018). Studies investigating the environmental impact of alternative diets, e.g., vegetarian, vegan, or flexitarian, have reported that dietary scenarios containing fewer animal products have lower environmental impacts than the current diets (Baroni et al., 2007; Friel et al., 2009; Risku‐Norja et al., 2008; Westhoek et al., 2014). However, meat and dairy products are high‐quality sources of protein and micronutrients, and ensuring their adequate bioavailability is important for public health (Aleksandrowicz et al., 2016). Animal products are an important provider of some essential nutrients (such as Fe, vitamin B12, and n‐3 fatty acids); therefore, restrictive and monotonous plant‐based diets can lead to nutrient deficiencies that have detrimental effects on health (McEvoy et al., 2012). Additionally, hypothetical dietary scenarios do not represent actual food consumption in terms of food options and energy content and ignore the cultural acceptability component of diet sustainability (Vieux et al., 2018). The results of this study underline that a reduction in environmental footprint can be achieved without completely removing animal foods from the diet. In light of this information, we think that it would be more sustainable to focus on providing an overall reduction in the environmental footprint of meal menus by reducing the amounts of food groups that contribute to the WF and GHGE the most, rather than eliminating these food groups, particularly red meat, and adopting a food group‐focused approach.

### 4.3 The changes in the environmental footprint of the state university hospital meal menus when planned according to the Turkey dietary guidelines nutritional model

There is a significant global shift in the nutritional preferences of consumers, especially in developing countries, from fruits, vegetables, and grains to meat, dairy products, and processed foods (FAO, 2018). It is emphasized that individuals' total WF (Capone et al., 2013; Chaudhary & Krishna, 2019; Harris et al., 2020; Jalava et al., 2014; Marlow et al., 2015; Springmann et al., 2018; Tompa et al., 2022) and GHGE (Drew et al., 2020; Gerber et al., 2013; Hyland et al., 2017; Kim & Neff, 2009; Temme et al., 2015; The World Bank, 2017; Vidal et al., 2015; Vieux et al., 2018) will decrease as their diets are transformed into healthier ones that comply with national nutrition guidelines. Tompa et al. (Tompa et al., 2022) evaluated individuals' current food consumption data and determined that planning meal menus according to the European Food Safety Authority and Hungarian dietary recommendations resulted in a decrease in the share of meat and meat products food group in the WF by 19.5% in women and 28.2% in men. A study conducted in New Zealand demonstrated that gradually reducing the animal protein content on the menu and increasing the vegetable protein content instead significantly reduced GHGE. Studies have reported that aligning current food consumption trends of the general population with national dietary guidelines can reduce GHGEs by 4%–42%, depending on the degree of alignment and minimization of food waste (Drew et al., 2020). In parallel with the studies in the literature, in this study, planning the standard meal menus of state university hospitals according to the national dietary guidelines resulted in a reduction of 24.8% and 31.7% in WF and GHGE, respectively (Figures 1 and 2).

### 4.4 The changes in the environmental footprint of the state university hospital meal menus when planned according to the Mediterranean nutritional model

There has been increased interest in the Mediterranean diet as a sustainable dietary model in the international literature on transitioning to more sustainable food systems and diets (Aboussaleh et al., 2017; Berry et al., 2015; Blas et al., 2019; Burlingame & Dernini, 2011; Capone et al., 2013; Dernini et al., 2013, 2017; Dernini & Berry, 2015; Shahar et al., 2023). The Mediterranean diet is considered a model for the development of sustainable diets as it has low environmental impacts, is rich in biodiversity, has a high socio‐cultural nutritional content, and has positive economic returns locally (Aboussaleh et al., 2017; Berry et al., 2015; Burlingame & Dernini, 2011; Dernini et al., 2013, 2017; Dernini & Berry, 2015). Capone et al. (Capone et al., 2013) reported that the WF of the current Italian diet is 69.9% higher than the recommended Mediterranean diet. Blas et al. (Blas et al., 2019) reported that the Mediterranean diet has better water‐nutrition efficiency since it provides more energy, fiber, and nutrients per liter of water consumed and that switching to the Mediterranean diet will reduce the WF by 750 L/person day. A study conducted in Spain reported that aligning the current diets in Spain with the Mediterranean diet decreased water consumption by 33% and GHGE by 72%, whereas aligning the current diets in Spain with the western‐style diet model increased all environmental footprints by between 12% and 72% (Sáez‐Almendros et al., 2013). In a study comparing different meal menus offered to hospital patients, Vidal et al. reported (Vidal et al., 2015) that the Mediterranean diet results in lower GHGE and that preparing guidelines for providing healthy food in hospitals worldwide is an effective step in combating climate change. In parallel with the literature, in this study, planning the meal menus of state university hospitals according to the Mediterranean nutritional model resulted in a reduction of 37.8% and 49.0% in WF and GHGE, respectively (Figures 1 and 2).

### 4.5 The strengths and limitations of the study

To the best of this study's authors' knowledge, in terms of sample size and number of menus evaluated, this study is the first in Turkey and one of the limited studies in the literature on the environmental footprint of hospital meal menus. A thorough review of the relevant literature revealed that the limited number of studies on the subject have addressed either the WF (Blas et al., 2016, 2019; Capone et al., 2013; Harris et al., 2020; Jalava et al., 2014) or GHGE (Drew et al., 2020; Gerber et al., 2013; Hyland et al., 2017; Vidal et al., 2015) of the hospital menus. In comparison, in this study, the environmental footprints of the hospital menus were investigated in terms of both WF and GHGE. We think that the findings of this study, which compares state university hospitals' standard meal menus and alternative menus based on TDG and Mediterranean nutritional models, will significantly contribute to the literature on sustainable nutrition. Self‐reported food consumption records can have a margin of error of up to approximately 22% due to under‐reporting of both total food intake and specific food types (Ravelli & Schoeller, 2020; Vidal et al., 2015). Minimizing this error by using food purchasing data and actual portion sizes in calculating the environmental footprint of foods on menus is another strength of this study. On the other hand, the limitations of this study were that the environmental footprint of food waste could not be calculated and that only state university hospitals were included in the study.

## 5 CONCLUSIONS

Considering the inseparable link between human and environmental health, it is imperative that hospitals, which are responsible for protecting and improving human health, also protect the environment. Hospitals serve millions of meals to patients and their families every year. Therefore, hospitals have a crucial role in both reducing the food‐related environmental footprint of healthcare services and promoting healthy and sustainable eating habits. The authors of this study believe that in order to reduce the environmental footprint of hospital food services in Turkey, the serving quantities and frequency of serving of food groups offered in hospitals should be clearly determined by the Ministry of Health. In this study, only the meal menus of state university hospitals were considered. However, this study's results may guide further studies that will include all hospitals. Ensuring a food supply process and elaborating menu planning policies that can provide both healthy nutrition and the lowest possible environmental burden can contribute to transitioning to a healthy and sustainable diet across the population.

## AUTHOR CONTRIBUTIONS

Conceptualization, B.O., M.K and E.K; Methodology, B.O. and M.K.; Software, M.K.; Validation, B.O, M.K. and E.K.; Formal Analysis, M.K.; Investigation, B.O; Resources, B.O.; Data Curation, M.K and E.K.; Writing – Original Draft Preparation, B.O; Writing – Review & Editing, M.K and E.K; Visualization, M.K; Supervision, B.O; Project Administration, B.O; Funding Acquisition, B.O.

## FUNDING INFORMATION

The authors declare that no funds, grants, or other support were received during the preparation of this manuscript.

## CONFLICT OF INTEREST STATEMENT

The authors have no relevant financial or non‐financial interests to disclose.

## ETHICS STATEMENT

This study was conducted in accordance with the principles outlined in the Declaration of Helsinki. The study protocol was approved by the Ethics Committee of Ağrı İbrahim Çeçen University (Approval Number: 346; Approval Date: November 25th, 2021) prior to the conduct of the study.

## Supporting information


Table S1

Table S2

Table S3

## Data Availability

Data will be made available on request.
