# Supplementary material for: Green hospitals: Mitigating water footprint and greenhouse gas emissions through sustainable menu planning in Turkish state university hospitals
Source: Food Sci Nutr. 2024 Jun 7;12(8):5966–78. doi: 10.1002/fsn3.4244 (PMC11317658; doi:10.1002/fsn3.4244)
Supplement: Supplementary file 1 — Table S1 [file FSN3-12-5966-s001.docx]

Supplementary Table 1. The features of standard hospital menus offered in hospitals in Turkey by the Inpatient Treatment Institutions Operation Regulation

| **Type of Food :** | **Daily Maximum Amount (g/piece)** | **Maximum Number of Gives per Week** | **How to use and replace** |
| --- | --- | --- | --- |
| Milk or Yogurt | 250 g | 7 | This milk or yoghurt can be used in meals when necessary. |
| Tea | 1.5 g | 7 | If necessary, it can be served with biscuits for afternoon breakfast. |
| Sugar | 20 g | 7 | For breakfasts |
| Sugar | 50 g | 5 | For desserts |
| Feta cheese | 40 g | 7 | For breakfast (20 g of cheddar or melted cheese can be given instead. |
| Feta cheese | 20 g | 2 | For pastry and pasta |
| Egg | 1 piece | 2 | For breakfasts |
| Egg | ½ piece | 2 | For pastries |
| Egg | ¼ piece | 4 | For soups |
| Jam or marmalade | 30 g | 7 | For breakfast (the same amount of molasses or honey can be given instead.) |
| Breakfast oil (Margarine) | 15 g | 3 | For breakfasts |
| Olive | 25 g | 4 | For breakfasts |
| Meat-Bone | 200 g | 7 | Instead of meat, 150 grams of chicken, turkey or fish can be given per meal. |
| Legumes | 50 g | 4 | For meals |
| Legumes | 15 g | 1 | For Ashura |
| Legumes - Flour or lentils | 20 g | 2 | For soups |
| Rice | 60 g | 3 | For pilafs (Bulgur can be used instead, not more than once.) |
| Rice | 20 g | 2 | For soups |
| Rice | 40 g | 3 | For stuffed vegetables |
| Rice | 10 g | 2 | For meatballs |
| Rice flour | 20 g | one | For desserts |
| Wheat | 15 g | one | For Ashura |
| Bulgur wheat | 60 g | one | Bulgur pilaf can be given once a week instead of rice pilaf. |
| Pasta | 60 g | one | The same amount of couscous can be given instead. |
| Noodle | 20 g | 2 | For soups |
| Barley Noodle | 10 g | 2 | To mix into rice |
| Tarhana | 20 g | one | For soup |
| Wheat flour | 50 g | one | For the pastry (75 g phyllo dough can be used instead) |
| Wheat flour | 50 g | one | For halva and dumplings |
| Wheat flour | 15 g | one | For flour soup |
| Wheat flour | 10 g | 2 | For marinating meatballs and soups |
| Starch | 20 g | one | For jelly |
| Semolina | 50 g | one | For halva and revani |
| Fresh vegetables | 350 g | 7 | Meals, salads, soups, side dishes (potatoes or 250 g canned vegetables can be used instead, not more than 2 times each) |
| Onion | 50 g | 7 | For meals and salads (12 g of spring onion can be given instead.) |
| Fresh fruits | 200 g | 4 | For fresh fruit, compote, berry, puree and juice |
| Melon/watermelon | 500 g | 4 | No recommendation |
| Dry fruit | 50 g | 2 | For fruit compote |
| Raisins | 10 g | one | For desserts |
| Dry fig | 10 g | one | For desserts |
| Tomato paste | 15 g | 7 | For meals |
| Parsley , mint, dill | 10 g | 7 | For meatballs, stuffed vegetables, wraps, salads and meals |
| Margarine | 40 g | 7 | For soups, stews and desserts |
| Vegetable oil or olive oil | 30 g | 7 | For vegetable dishes, stuffed salads and wraps |
| Walnut | 10 g | 3 | For desserts |
| Hazelnut | 10 g | one | For desserts |
| Spices | 0.5 g | 3 | For meals |
| Salt | 20 g | 7 | For meals |
