# Supplementary material for: Green hospitals: Mitigating water footprint and greenhouse gas emissions through sustainable menu planning in Turkish state university hospitals
Source: Food Sci Nutr. 2024 Jun 7;12(8):5966–78. doi: 10.1002/fsn3.4244 (PMC11317658; doi:10.1002/fsn3.4244)
Supplement: Supplementary file 2 — Table S2 [file FSN3-12-5966-s003.docx]

|  | **Food Groups** | **R1** | **R2** | **R3** | **R4** | **R5** | **R6** | **R7** | **R8** | **R9** | **R10** | **R11** | **R12** | **AOR** | **TDG** | **MED** |
| --- | --- | --- | --- | --- | --- | --- | --- | --- | --- | --- | --- | --- | --- | --- | --- | --- |
| **Animal Origin** | **Milk and Dairy Products** | 8121,36 | 7284 | 8072,8 | 8126,3 | 8113,2 | 8065,48 | 8327,92 | 8185 | 8360,72 | 7797 | 7647 | 7138 | 7936,57 | 15457,47 | 10383,01 |
|  | **Read Meat** | 2806,83 | 2494,11 | 3432,78 | 2937,58 | 3507,8 | 3503,9 | 3012,98 | 3290,08 | 3283,49 | 3264,3 | 3015,09 | 3410,55 | 3163,29 | 1325,38 | 764,8 |
|  | **Poultry** | 2330,09 | 2483,21 | 2265,35 | 2301,72 | 2240 | 2518,3 | 1833,86 | 2335,43 | 2041,65 | 2256,8 | 2193,75 | 936 | 2144,68 | 1366,85 | 918,18 |
|  | **Eggs** | 688,42 | 872,38 | 1014,57 | 698,78 | 656,7 | 886,16 | 930,29 | 1286,19 | 611,53 | 705,1 | 984,72 | 599,13 | 827,83 | 987,98 | 1201,64 |
| **Vegetable Origin** | **Cereals** | 3653,57 | 3946,17 | 4511,54 | 4498,82 | 4701,42 | 4053,28 | 4680,23 | 4957,65 | 3775,17 | 3817,54 | 3675,93 | 3425,53 | 4141,4 | 6750,93 | 5808,52 |
|  | **Legumes and Pulses** | 959,38 | 840,27 | 964,92 | 1222,85 | 1127,28 | 1052,92 | 924,21 | 940,18 | 995,96 | 915,44 | 1001,88 | 617,76 | 963,59 | 992,32 | 808,75 |
|  | **Vegetables** | 16678,23 | 16864,54 | 19399,06 | 17219,89 | 18196,79 | 16008,52 | 17102,05 | 18070,05 | 16554,54 | 16137,68 | 16044,87 | 16189,57 | 17038,82 | 14807,05 | 26910,95 |
|  | **Fruits** | 3811,79 | 4003,31 | 4163,39 | 4501,1 | 4382,69 | 4431,84 | 5005,49 | 7240,19 | 5456,2 | 5230,44 | 5099,95 | 5160,08 | 4873,87 | 10604,41 | 15280,62 |
| **Oils and fats** | **Oils and fats** | 1230,96 | 1224,52 | 1138,44 | 1701,37 | 1142,33 | 1719,78 | 1155,14 | 1350,66 | 1044,7 | 1204,52 | 1009,4 | 1017,03 | 1244,9 | 2863,17 | 4790,38 |
| **Others** | **Nuts** | 144,38 | 193,15 | 155,85 | 80,55 | 155,89 | 147,49 | 184,8 | 308,29 | 146,47 | 147,71 | 144,8 | 121,53 | 160,91 | 418,74 | 709,95 |
|  | **Sugar** | 440,08 | 684,23 | 601 | 639,63 | 481 | 702,53 | 628,98 | 535,95 | 393,42 | 533,75 | 567 | 582,67 | 565,85 | 0 | 0 |
|  | **Others** | 102,84 | 463,07 | 83,6 | 87,45 | 105,6 | 233,81 | 93,6 | 99,1 | 67,32 | 78,8 | 69,3 | 89,93 | 131,2 | 480,01 | 760,12 |

Supplementary Table 2. Detailed information about the food groups and quantities (g/month) of the hospital menus, TDG and MED

R:Regions; AOR:Average of Regions; TDG:Turkey Dietary Guidelines; MED:Mediterranean Diet
