# Supplementary material for: Green hospitals: Mitigating water footprint and greenhouse gas emissions through sustainable menu planning in Turkish state university hospitals
Source: Food Sci Nutr. 2024 Jun 7;12(8):5966–78. doi: 10.1002/fsn3.4244 (PMC11317658; doi:10.1002/fsn3.4244)
Supplement: Supplementary file 3 — Table S3 [file FSN3-12-5966-s002.docx]

Supplementary Table 3. The WF and GHGE values of hospital menus and dietary models

|  | **Green WF (L/month)** | **Blue WF**  **(L/month)** | **Gray WF**  **(L/month)** | **Total WF**  **(L/month)** | **GHGE**  **(kg CO_2_-eq/month)** |
| --- | --- | --- | --- | --- | --- |
| **R1** | 106172 | 10352.9 | 8062.73 | 124588 | 128.7 |
| **R2** | 117142 | 23141.8 | 8977.48 | 149261 | 137.6 |
| **R3** | 120682 | 11403.5 | 8823.89 | 140909 | 146.3 |
| **R4** | 114198 | 10621 | 8653.07 | 133472 | 134.7 |
| **R5** | 132979 | 12420.8 | 9673.53 | 155074 | 161.3 |
| **R6** | 137155 | 12230.5 | 9869.64 | 159255 | 167.3 |
| **R7** | 111161 | 11054.3 | 8385.03 | 130599 | 134.6 |
| **R8** | 127739 | 12678.4 | 9668.9 | 150087 | 153.4 |
| **R9** | 114163 | 10506.4 | 8227.09 | 132896 | 140.5 |
| **R10** | 117381 | 10829.8 | 8507.06 | 136718 | 140.5 |
| **R11** | 105483 | 9834.53 | 7721.21 | 123039 | 127.4 |
| **R12** | 106606 | 9832.21 | 7353.55 | 123792 | 136.8 |
| **AOR** | 115757 | 12941.7 | 8581.86 | 137280 | 139.9 |
| **TDG** | 84299.8 | 10557.8 | 8244.45 | 103207 | 95.5 |
| **MED** | 66797.2 | 10713.6 | 7932.9 | 85420.5 | 71.3 |
